# Apolipoprotein E-dependent load of white matter hyperintensities in Alzheimer’s disease: a voxel-based lesion mapping study

**DOI:** 10.1186/s13195-015-0111-8

**Published:** 2015-05-15

**Authors:** Katrin Morgen, Michael Schneider, Lutz Frölich, Heike Tost, Michael M Plichta, Heike Kölsch, Fabian Rakebrandt, Otto Rienhoff, Frank Jessen, Oliver Peters, Holger Jahn, Christian Luckhaus, Michael Hüll, Hermann-Josef Gertz, Johannes Schröder, Harald Hampel, Stefan J Teipel, Johannes Pantel, Isabella Heuser, Jens Wiltfang, Eckart Rüther, Johannes Kornhuber, Wolfgang Maier, Andreas Meyer-Lindenberg

**Affiliations:** Central Institute of Mental Health, Medical Faculty Mannheim/Heidelberg University, J5, 68159, Mannheim, Germany; Institute of Human Genetics, University of Bonn, Sigmund-Freud-Str. 25, 53127, Bonn, Germany; Department of Medical Informatics, University of Göttingen, Robert-Koch-Str. 40, 37075, Göttingen, Germany; Department of Psychiatry and Psychotherapy, University of Bonn, Bonn, Germany; Department of Psychiatry and Psychotherapy, Charité - Universitätsmedizin Berlin, Hindenburgdamm 30, 12203, Berlin, Germany; Department of Psychiatry and Psychotherapy, University of Hamburg, Martinistr. 52, 20246, Hamburg, Germany; Department of Psychiatry and Psychotherapy, University of Düsseldorf, Bergische Landstr. 2, 40629, Düsseldorf, Germany; Department of Psychiatry and Psychotherapy, University of Freiburg, Hauptstr. 5 79104, Freiburg, Germany; Department of Psychiatry and Psychotherapy, University of Leipzig, Semmelweisstr. 10, 04103, Leipzig, Germany; Department of Psychiatry and Psychotherapy, University of Heidelberg, Voßstr. 5, 69115, Heidelberg, Germany; Département de Neurologie, Institut de la Mémoire et de la Maladie d’Alzheimer, Hôpital de la Salpêtrière Paris, Université Pierre et Marie Curie, 47 Blvd. de lHopital, 75013, Paris, France; Department of Psychiatry and Psychotherapy, University of Rostock and DZNE Rostock, Gehlsheimerstr. 20, 18147 Rostock, Rostock, Germany; Institute of General Practice, University of Frankfurt, Theodor-Stern-Kai 7, 60590, Frankfurt, Germany; Department of Psychiatry and Psychotherapy, University of Essen, Virchowstr. 174, 45147, Essen, Germany; Department of Psychiatry and Psychotherapy, University of Göttingen, Von-Siebold-Str. 5, 37075, Göttingen, Germany; Friedrich-Alexander-University Erlangen-Nuremberg, Schwabachanlage 6, 91054, Erlangen, Germany; German Center for Neurodegenerative Diseases (DZNE), Holbeinstr. 13-15, 53175, Bonn, Germany; AHG-Klinik für Psychosomatik, Kurbrunnenstr. 12, 67098, Bad Dürkheim, Germany

## Abstract

**Introduction:**

White matter (WM) magnetic resonance imaging (MRI) hyperintensities are common in Alzheimer’s disease (AD), but their pathophysiological relevance and relationship to genetic factors are unclear. In the present study, we investigated potential apolipoprotein E (*APOE*)-dependent effects on the extent and cognitive impact of WM hyperintensities in patients with AD.

**Methods:**

WM hyperintensity volume on fluid-attenuated inversion recovery images of 201 patients with AD (128 carriers and 73 non-carriers of the *APOE* ε4 risk allele) was determined globally as well as regionally with voxel-based lesion mapping. Clinical, neuropsychological and MRI data were collected from prospective multicenter trials conducted by the German Dementia Competence Network.

**Results:**

WM hyperintensity volume was significantly greater in non-carriers of the *APOE* ε4 allele. Lesion distribution was similar among ε4 carriers and non-carriers. Only ε4 non-carriers showed a correlation between lesion volume and cognitive performance.

**Conclusion:**

The current findings indicate an increased prevalence of WM hyperintensities in non-carriers compared with carriers of the *APOE* ε4 allele among patients with AD. This is consistent with a possibly more pronounced contribution of heterogeneous vascular risk factors to WM damage and cognitive impairment in patients with AD without *APOE* ε4-mediated risk.

**Electronic supplementary material:**

The online version of this article (doi:10.1186/s13195-015-0111-8) contains supplementary material, which is available to authorized users.

## Introduction

White matter (WM) hyperintensities (WMHs) resulting from small vessel vasculopathy are commonly observed on T2-weighted magnetic resonance imaging (MRI) scans of elderly persons [1]. In patients with Alzheimer’s disease (AD), WMH load has been reported to be increased compared with demographically similar subjects without dementia [2-10], in line with strong neuropathological evidence that cerebrovascular disease is more common in AD than in synucleinopathies and frontotemporal dementia or in the absence of neurodegenerative disease [11].

The pathogenesis of WM damage in AD is likely to be multifactorial and to involve non-specific vascular risk factors as well as endothelial injury mediated by amyloid deposition [12]. Vascular risk factors such as hypertension are known to increase both the prevalence and progression of WMHs [13], as well as of AD microscopic lesions, such as amyloid plaques and neurofibrillary tangles [14]. Recently, a significant contribution of AD-specific mechanisms to WMHs was indicated in a prospective cohort derived from the Baltimore Longitudinal Study of Aging Autopsy Program [15]. In that study, several measures of AD pathology, such as Braak score and composite AD pathology score, correlated with WMH volume at autopsy [15]. Furthermore, patients diagnosed with amyloid angiopathy show an accelerated progression of WMH volume [7,16]. Conversely, there is evidence that WMHs contribute to the risk of AD largely independently of cerebral amyloid-β (Aβ) deposition, suggesting that potentially heterogeneous WM damage may lower the threshold for a diagnosis of AD in the presence of amyloid pathology [17].

Depending on their location and severity, WM lesions can affect various cognitive domains by disrupting fiber tract integrity or prompting retrograde neuronal degeneration. Although WMH increase the risk of global cognitive decline (for example [18]), cognitive functions most consistently impaired by disseminated subcortical and periventricular WM damage are speed of information processing and executive function [5,7,19-21].

To what extent the mechanisms of WM damage relate to genetic factors remains unclear. The purpose of the present study was to examine potential apolipoprotein E (*APOE*)-dependent effects on the distribution and cognitive impact of WMHs in patients with AD. We hypothesized that if WMHs indicate a separate vascular aspect of AD pathology, they should be increased in *APOE* ε4 risk allele non-carriers, whereas the opposite prediction would be made if WMHs predominantly mediate genetic risk of *APOE*.

## Methods

### Ethics statement

The study was approved by the Central Institutional Review Board (IRB) of the German Dementia Network located at the University of Erlangen and by each of the local IRBs of the participating centers (that is, the IRBs of Charité - Universitätsmedizin Berlin and the medical faculties of the universities of Bonn, Erlangen-Nuremberg, Freiburg, Göttingen, Hamburg and Heidelberg and Ludwig-Maximilians-University Munich). All subjects gave their informed consent to participate in the study.

### Subjects

Two hundred one patients who fulfilled the National Institute of Neurological and Communicative Disorders and Stroke–Alzheimer’s Disease and Related Disorders Association (NINCDS-ADRDA) criteria of probable AD [22] (128 carriers of the *APOE* ε4 allele and 73 non-carriers) were included in the study. Among the 128 carriers of the ε4 allele, 39 were homozygous (19.4% of sample overall). Of the 201 patients, 168 (113 ε4 carriers and 55 non-carriers) had a diagnosis of probable AD [22] when the data were collected, 32 subjects (15 ε4 carriers and 17 non-carriers) had mild cognitive impairment (MCI) at the time of analysis and converted to AD within the following 1.4 ± 0.6 years (ε4 carriers: 1.3 ± 0.5 years, non-carriers: 1.5 ± 0.7 years). Only 15 of 201 subjects were carriers of the ε2 allele (7.5%). Because of lack of power, effects of ε2 carrier status were not investigated.

The NINDS-ADRDA criteria lack precise guidelines on how to consider “silent” vascular lesions in the case of an AD-typical clinical course of dementia [22]. According to the new diagnostic recommendations of the National Institute on Aging-Alzheimer’s Association workgroup, patients should not be diagnosed with probable AD in the “presence of multiple or extensive infarcts or severe white matter hyperintensity burden” [23] (p. 266). Severe WMH burden, in turn, is classified as hyperintensity volume on MRI greater than 25% of WM and thus considered indicative of vascular dementia on the basis of the National Institute of Neurological Disorders and Stroke–Association Internationale pour la Recherche et l’Enseignement en Neurosciences (NINDS-AIREN) criteria [24,25]. To achieve high specificity for AD in the present study, we excluded patients with MRI evidence of severe cerebrovascular disease according to the new AD diagnostic guidelines and NINDS-AIREN criteria [23-25]—that is, with strategic territorial and cortical watershed infarctions or extensive small vessel disease defined by multiple lacunar infarctions, bilateral thalamic lesions or greater than 25% WMH burden. Thus, a threshold greater than 10 cm^3^ was set, which has previously been applied to define severe disseminated WM cerebrovascular disease [26,27] and has been found to approximate 25% of WM [28]. A threshold of 10 cm^3^ also distinguishes subjects with severe (that is, grade 3) WMH burden from subjects with less pronounced WM damage according to the well-established semiquantitative Fazekas scale [26,28,29]. As a result, 18 patients (eight ε4 carriers and ten non-carriers) were excluded on the basis of lesion volume greater than 10 cm^3^. In order to detect a potential bias due to exclusion of subjects with severe lesion burden, we investigated characteristics of this subgroup and also repeated the analysis of *APOE*-dependent effects for the entire group of 201 subjects.

The characteristics of the 183 patients with AD (120 ε4 carriers and 63 non-carriers) included in the main analysis according to the new AD diagnostic guidelines and NINDS-AIREN criteria [23-25] are listed in Tables 1 and 2. The characteristics of the subgroup (n = 18) excluded from the main analysis because of severe lesion volume and of the entire group (n = 201) are presented in Table 2 (WMH data), Additional file 1: Table S1 and Additional file 2: Table S2 (demographic and clinical variables). Clinical evaluation of patients consisted of a complete neurological and psychiatric examination. Cognitive status was assessed with the Mini Mental State Examination (MMSE) and the Clinical Dementia Rating (CDR) scale. Global CDR score and CDR Sum of Boxes (CDR SOB) were determined; the latter was assessed by assigning a severity score in six domains (memory, orientation, judgment and problem solving, community affairs, home and hobbies). CDR SOB scores show greater variability than global CDR scores. Current diagnoses of diabetes (based on fasting glucose levels ≥7 mmol/L or treatment), hypertension (based on systolic blood pressure >140 mmHg or diastolic blood pressure >90 mmHg or antihypertensive medication), treatment with cholesterol-lowering medication and self-reported coronary heart disease were assessed in the majority of subjects (Table 1, Additional file 1: Table S1 and Additional file 2: Table S2).

**Table 1:** **Characteristics of patients with probable Alzheimer’s disease according to new diagnostic guidelines** ^**a**^

	***APOE *** **ε4 carriers (n = 120)**	***APOE*** **ε4 non-carriers (n = 63)**	**Group comparison** ***P*** **-values** ^**b**^
Total number of patients	N = 183		
Age (yr)	70.4 ± 6.4	70.4 ± 8.7	0.97
Age at onset (yr)	67.6 ± 7.2	66.8 ± 12.2	0.96
Duration of disease (mo)	31.3 ± 24.7	30.5 ± 25.4	0.84
Males/females, n (ratio)	57/63 (1:1.1)	28/35 (1:1.3)	0.70
Education (yr)	9.1 ± 1.8	9.4 ± 2.2	0.30
Systolic blood pressure (mmHg)	139.3 ± 17.0^c^	141.1 ± 16.9^d^	0.54
Systolic blood pressure ≥140 mmHg (yes/no)	57/42 (1.4 : 1)	32/23 (1.4 : 1)	0.95
Diastolic blood pressure (mmHg)	81.6 ± 8.7^c^	83.8 ± 7.9^d^	0.14
Diastolic blood pressure ≥90 mmHg (yes/no)	33/66 (1:2)	20/35 (1:1.8)	0.78
Antihypertensive medication (yes/no)	31/87 (1 : 2.8)^e^	20/39 (1 : 2)^f^	0.18
Coronary heart disease (yes/no)	7/111 (1 : 15.9)^e^	5/56 (1 : 9.3)^g^	0.57
Diabetes (yes/no)	16/102 (1 : 6.4)^e^	6/53 (1 : 8.8)^f^	0.45
Hypercholesterolemia (yes/no)	15/100 (1 : 6.7)^h^	7/47 (1 : 6.7)^i^	0.99
BMI	24.5 ± 3.9^j^	24.2 ± 4.9^i^	0.85
CDR SOB	4.3 ± 1.4	4.3 ± 1.6	0.92
MMSE (score)	23.8 ± 3.2^k^	24.5 ± 2.9	0.51
Delayed verbal recall (score)	2.1 ± 2.0^j^	2.8 ± 2.2	0.02
Verbal learning	12.1 ± 4.3^j^	12.4 ± 4.4	0.87
Trail Making Test A (s)	96.6 ± 57.0^l^	96.1 ± 52.3	0.60
Constructive -apraxia	9.0 ± 2.3^k^	9.0 ± 1.8	0.59
Boston Naming Test	12.6 ± 2.4^k^	12.8 ± 2.4	0.80

^a^ *APOE*, Apolipoprotein E; BMI, Body mass index; CDR SOB, Clinical Dementia Rating Sum of Boxes; MMSE, Mini Mental State Examination. Data are presented as mean ± SD or ratio. ^b^ *P*-values are based on Student’s *t*-test. Available data: ^c^n = 99, ^d^n = 55, ^e^n = 118, ^f^n = 59, ^g^n = 61, ^h^n = 115, ^i^n = 54, ^j^n = 99, ^k^n = 119, ^l^n = 116.

**Table 2:** **White matter hyperintensity characteristics** ^**a**^

	**Mean, mm** ^**3**^	**SD**	**SE**	**95% confidence interval**		**Group comparison** ***P*** **-value**
				**Lower**	**Upper**	
Patients with white matter hyperintensity (WMH) volume ≤10 cm^3^ (n = 183)						
*APOE* ε4 carriers (n = 120)	1,857	2,026	185	1,491	2,223	0.01
*APOE* ε4 non-carriers (n = 63)	2,873	2,780	350	2,173	3,573	
Patient group overall (including subjects with severe WMH volume, n = 201)						
*APOE* ε4 carriers (n = 128)	2,659	3,810	337	1,993	3,325	0.01
*APOE* ε4 non-carriers (n = 73)	4,940	6,616	774	3,396	6,483	

^a^ *APOE*, Apolipoprotein E; SD, Standard deviation; SE, Standard error.

We used data collected from prospective multicenter trials conducted by the German Dementia Competence Network [30]. The study cohort was identified retrospectively from among these trial subjects. Patients included in the present study were recruited in eight German centers. Additional inclusion criteria were the availability of neuropsychological test results, *APOE* genotyping, a high-resolution three-dimensional fast T1-weighted gradient echo sequence and a fluid-attenuated inversion recovery (FLAIR) sequence. Furthermore, data were included only after quality control of the MRI scans, which consisted of a test of image homogeneity covariance and noise estimation using voxel-based morphometry (VBM) with the VBM8 toolbox [31] as well as visual inspection. Seven patients had to be excluded because of motion or susceptibility artifacts. Other exclusion criteria were stroke, motor symptoms associated with other neurodegenerative diseases such as Lewy body dementia, and cognitive impairment secondary to recognizable diseases such as head injury, multiple sclerosis or normal pressure hydrocephalus. In addition, subjects with clinically relevant depression, defined as a score of 4 or more on the depressive symptom subscale of the Neuropsychiatric Inventory (NPI) [32], were excluded.

### Neuropsychological testing

The neuropsychological battery included immediate and delayed recall of word lists, the Boston Naming Test (a test of word retrieval), drawing of increasingly complex figures (constructional praxis) and free recall of drawings from the cognitive battery designed by the Consortium to Establish a Registry for Alzheimer’s Disease [33]. Subjects were also assessed with the Trail Making Test (TMT) parts A and B, which are sensitive to speed of information processing, mental flexibility and executive function. Because of floor effects, results for the TMT B were not included in further analyses. Performance on the TMT A and the delayed verbal recall task were selected for analyses of correlations with MRI measures of tissue damage. TMT A performance was chosen for further analysis because of its established association with disseminated WM damage [34], and the delayed verbal recall task was selected because of its particular sensitivity to AD pathology.

### Structural image parameters

MRI examinations were conducted using 1.5-T whole-body units. Siemens scanners (MAGNETOM Vision, Symphony or Sonata; Siemens Healthcare, Erlangen, Germany) were used at six centers, and Philips scanners (Gyroscan Intera; Philips Medical Systems, Eindhoven, Netherlands) were employed at the remaining two centers. T1-weighted scanning was performed with a sagittal magnetization prepared rapid gradient echo sequence on the Siemens scanners and a three-dimensional fast T1-weighted gradient echo sequence on the Philips scanners. The repetition time (TR) varied between 9.3 and 20 milliseconds, and the echo time (TE) between 3.93 and 4.38 milliseconds, between centers. The flip angle was approximately 15°, slice thickness from 1 to 1.2 mm, matrix between 256 × 256 pixels and 512 × 512 pixels, and field of view between 250 × 250 mm and 300 × 300 mm. FLAIR images were obtained with TE ranging from 100 to 110 milliseconds and TR from 9,000 to 10,000 milliseconds between centers. Inversion recovery time was 2,500 milliseconds. Images were two-dimensional with a slice thickness between 5 and 6 mm, matrix between 204 × 256 pixels and 220 × 512 pixels, and field of view between 191 × 240 mm and 256 × 256 mm.

### Lesion probability maps

Lesion maps were automatically calculated for each subject with the Lesion Segmentation Toolbox (LST) [35], an extension of the VBM8 toolbox [36], implemented within SPM8 (Statistical Parametric Mapping; Wellcome Trust Centre for Neuroimaging, London, UK [37]) and MATLAB version 8 software (MathWorks, Natick, MA, USA). Individual FLAIR images were corrected for MRI field inhomogeneity and coregistered to the respective T1-weighted images. Each voxel of the individual native T1-weighted image was assigned to gray matter (GM), WM or cerebrospinal fluid (CSF). Based on the tissue specific FLAIR intensity values, the LST algorithm derives an initial lesion map by identifying hyperintense outliers as potential lesions. Using a Markov random fields–based lesion-growing algorithm, the final lesion maps are computed in an iterative process.

### Voxel-based morphometry with T1-weighted magnetic resonance imaging

Processing of high-resolution T1-weighted images was based on the unified segmentation model [38] and conducted with SPM8 and MATLAB version 8 software. The method incorporates an iterated scheme combining bias correction; segmentation into WM, GM and CSF; and registration of prior images to stereotactic space. During the normalization procedure, images were interpolated to isotropic 1 × 1 × 1-mm voxels. The VBM8 toolbox was used to extend this model with a partial volume estimation and the application of a spatially adaptive non-local means filter [39] for bias correction. During normalization to stereotactic space, linear affine registration and linear deformation corresponding to a high-dimensional DARTEL normalization [40] were performed as implemented in VBM8. GM probability maps were then modulated (that is, intensity-corrected for local volume changes during normalization) to increase their sensitivity to the distribution of GM and WM volume, followed by smoothing with a 12-mm full width at half-maximum kernel.

### *APOE* ε4 genotyping

*APOE* genotyping involved isolation of leukocyte DNA with the Qiagen blood isolation kit according to the instructions of the manufacturer (Qiagen, Hilden, Germany). Subsequently, the presence of ε2, ε3 and/or ε4 alleles was determined using restriction isotyping by gene amplification and *Hha*I cleavage as described by Hixson and Vernier [41].

### Statistical analysis

#### *Effects of* APOE *ε4 genotype status on white matter hyperintensity*

To test the hypothesis that *APOE* ε4 status is related to WMH, we estimated an analysis of variance (ANOVA) model with *APOE* ε4 status (*APOE* ε4 non-carrier versus *APOE* ε4 carrier) as the factor of interest and age, sex, education level, disease classification and total intracranial volume (determined on T1-weighted MRI in native space, that is, prior to normalization) as covariates (Table 2 and Additional file 3: Table S3, model 1).

For a limited number of subjects with available data (n = 129 with WMH volume ≤10 cm^3^), a second model was specified that also accounts for a variety of vascular risk factors, disease duration and MMSE performance (Table 2 and Additional file 3: Table S3, model 2).

To assess the robustness of our findings, we also analyzed group differences between *APOE* ε4 carriers and non-carriers with the non-parametric Mann–Whitney *U* test.

*APOE* effects on WMH volume were investigated in a binary fashion (presence of at least one ε4 allele versus absence of ε4). In a secondary analysis, effects on WMH load were investigated with regard to ε4 dose (ε4 homozygosity or heterozygosity or absence of ε4 allele) (Additional file 4: Table S4).

To control for potential center effects on MRI measures, centers were included as additional covariates in all analyses involving WMH volume (SPSS for Windows, Version 22.0.0, 2013; IBM, Armonk, NY, USA).

#### Cognitive impact of white matter hyperintensities

To test if WMH is associated with executive functioning and speed of information processing, functions typically impaired by disseminated WM damage (for example, see [34]), we estimated an ANOVA model with TMT A performance as the dependent variable and age, sex, education level, disease classification, total intracranial volume, *APOE* ε4 status, categorical variables for centers and WMH volume as covariates.

#### White matter hyperintensity distribution

The statistical analysis of lesion distribution was performed with the non-parametric mapping module in MRIcron (version 7/2012; http://www.nitrc.org/frs/?group_id=152) [42]. To investigate potential group differences between carriers and non-carriers of the *APOE* ε4 allele, non-parametric Brunner-Munzel tests were conducted. A permutation-based threshold of *P* < 0.05 was chosen (1,000 permutations). The analysis was limited to voxels classified as hyperintensities in a minimum of 15% of the sample (n = 27).

#### Regional gray matter volume

SPM8 was used to analyze group differences with one-way ANOVA and to investigate effects on neuropsychological performance with multiple regression analyses. Age, sex, education level, total intracranial volume, center and stage of disease (MCI or dementia) were included as confounding variables on a voxel-by-voxel basis. Effects were reported as significant when they exceeded a conservative whole-brain voxel-level family-wise error (FWE)–corrected threshold of *P* < 0.05.

Additional region of interest (ROI) analyses were performed with *P* < 0.05 set as the voxel-level FWE-corrected significance level for the hippocampus and the prefrontal and posterior parietal cortices, based on previous findings of *APOE*-dependent volume effects in patients with early AD [43,44]. For this purpose, hippocampal and posterior parietal and superior frontal masks were created with the Harvard-Oxford probabilistic atlas of human cortical and subcortical areas [45]. Masks were visually inspected.

## Results

### Sample characteristics

As indicated in Table 1 and Additional file 2: Table S2, *APOE* ε4 carriers and non-carriers were well balanced with respect to a wide range of demographic and clinical variables. With the exception of delayed verbal recall (*P* = 0.02), we did not find any significant group differences for neuropsychological measures, suggesting that both groups were at comparable stages of dementia. Thus, these variables are unlikely to have confounded observed differences in WMH volume.

### White matter hyperintensity volume

The data show significantly lower WMH volumes in *APOE* ε4 carriers than in non-carriers (Tables 2 and 3, Additional file 3: Table S3). As evidenced by our extended statistical model (model 2), (n = 129), this effect cannot be explained by vascular risk factors or the status of disease as captured by disease duration, classification as MCI or dementia and MMSE performance (Table 3, Additional file 3: Table S3). The significant difference in WMH volume between *APOE* ε4 carriers and non-carriers was confirmed by the non-parametric Mann–Whitney *U* test (*P* < 0.02 for patients with WMH ≤10 cm^3^ (n = 183) and *P* < 0.01 including subjects with higher WMH (n = 201)).

**Table 3:** **Effects on total white matter hyperintensity volume (analysis of variance model with** ***APOE*** **ε4 status (non-carrier versus carrier as factor of interest) in subjects with white matter hyperintensity** ^**a**^ **≤10 cm** ^**3**^

	**Model 1 (n = 183)**		**Model 2 (n = 129)**	
	***f*** **(1, 169)**	***P*** **-value**	***f*** **(1, 107)**	***P*** **-value**
*APOE* ε4 carrier status	8.9	0.01	9.1	0.01
Age	39.7	0.01	42.6	0.01
Sex	1.5	0.22	2.4	0.13
Education	0.69	0.41	0.03	0.86
Disease classification	1.4	0.24	1.7	0.20
Total intracranial volume	4.9	0.03	9.2	0.01
Duration of disease			0.07	0.80
MMSE score^b^			0.004	0.95
Systolic blood pressure			0.16	0.69
Diastolic blood pressure			10.4	0.01
Antihypertensive medication			0.40	0.53
Coronary heart disease			0.004	0.95
Cholesterol medication			0.49	0.49
Diabetes medication			0.006	0.94

^a^Results for site covariates are not reported. ^b^MMSE, Mini Mental State Examination.

Elevated diastolic blood pressure had a significant impact on WMH volume. Other vascular risk factors, disease duration and MMSE performance were not significantly related to WMH volume (Table 3, Additional file 3: Table S3).

When subjects were classified according to dose of the ε4 allele (homozygosity for ε4, heterozygosity, absence of ε4 allele), *APOE*-dependent effects on WMH volume remained significant (Additional file 4: Table S4) and were attributable to the difference between ε4 carriers and non-carriers. WMH load did not differ between heterozygous and homozygous carriers of the ε4 allele (model 1: *f*(1, 106) = 0.31, *P* = 0.58; model 2: *f*(1, 64) = 0.26, *P* = 0.61).

### Distribution of white matter hyperintensity

**Figure 1:**
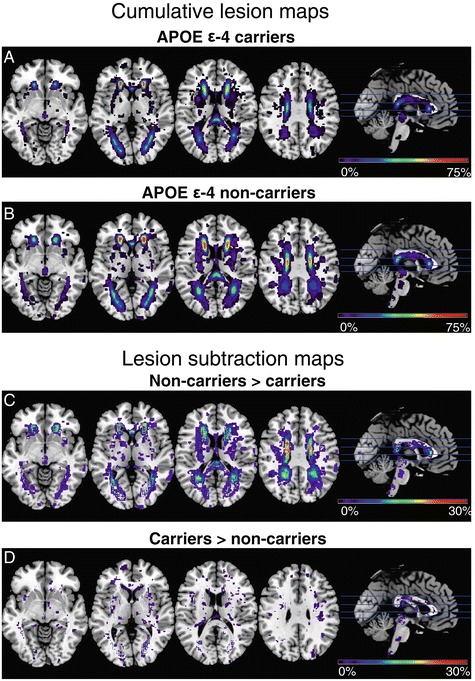
**Apolipoprotein E-dependent lesion probability distributions. (A)** Cumulative lesion maps in 120 carriers of the apolipoprotein E (*APOE*) ε4 allele. **(B)** Cumulative lesion maps in 63 non-carriers of the *APOE* ε4 allele. Note that the color scale indicates minimum to maximum overlap of lesions in Montreal Neurological Institute space as percentage of group size. **(C)** and **(D)** Lesion subtraction maps. The cumulative lesion maps are subtracted from each other without a statistical threshold to allow a direct comparison of lesion probability distributions.

**Figure 2:**
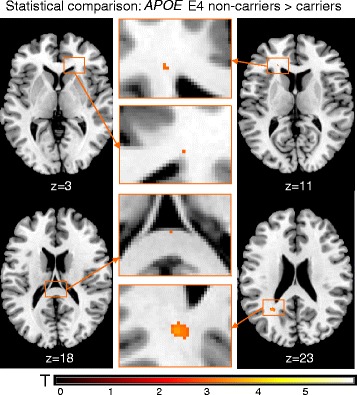
**Voxel-based statistical group comparison (non-carriers > carriers).** In non-carriers of the apolipoprotein (*APOE*) ε4 allele, lesions were more likely to occur at the horns of the lateral ventricles and the splenium of the corpus callosum than they were in carriers at a whole-brain permutation-based threshold of *P* < 0.05 (z = 3.0).

Cumulative WMH maps (Figure 1A, B) and lesion subtraction maps (Figure 1C, D) indicated that WMHs were distributed similarly among ε4 carriers and non-carriers. On the basis of voxel-wise permutation testing, differences in lesion volume reached statistical significance proximate to both anterior horns and the left posterior horn of the lateral ventricles as well as the splenium of the corpus callosum. The largest cluster was located near the posterior horn of the left lateral ventricle (maximum z-value 3.82, threshold at 3.00) (Figure 2). Lesion density was highest in the periventricular regions in both groups (Figure 1A, B).

### Gray matter volume distribution

**Figure 3:**
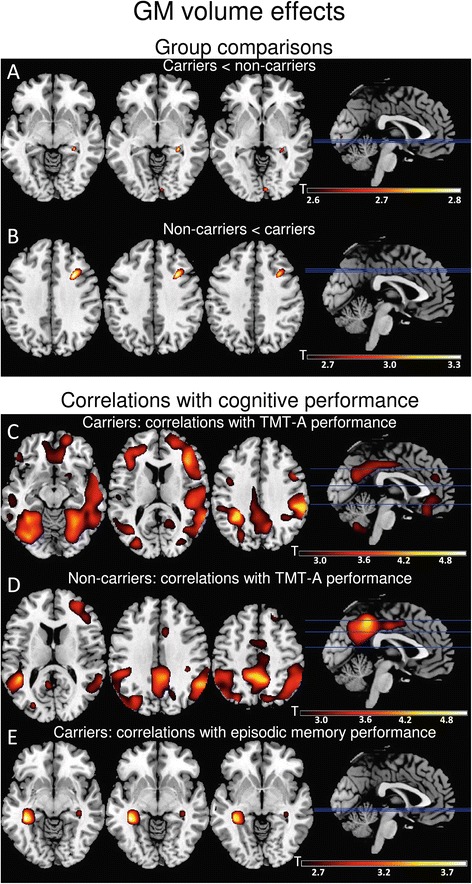
**Gray matter volume effects. (A)** and **(B)** Apolipoprotein (*APOE*)-dependent gray matter (GM) distribution. Carriers exhibited a tendency toward more hippocampal atrophy (A), whereas non-carriers showed a tendency toward more prefrontal volume loss (B). **(C)**, **(D)** and **(E)** Correlations between GM volume and cognitive performance. In carriers of the *APOE* ε4 allele, performance on the Trail Making Test part A (TMT-A) correlated with GM volume in the right frontal region as well as the bilateral temporal and parietal regions (C). Correlations with TMT-A performance in non-carriers occurred in the bilateral temporal and parietal regions (D). Carriers exhibited a correlation between delayed verbal recall performance and hippocampal volume (E), whereas recall performance in non-carriers did not correlate with GM volume. The results are presented at *P* < 0.005 for whole brain, uncorrected.

Carriers of the ε4 allele showed a tendency toward reduced volume in the right hippocampus compared with non-carriers, whereas ε4 non-carriers exhibited a tendency toward decreased volume in the right superior frontal gyrus compared with carriers (Table 4, Figure 3A and 3B).

**Table 4:** **Regional differences in brain volume between apolipoprotein E ε4 carriers and non-carriers** ^**a**^

**Location**	**MNI coordinates**			**z-value**	**Voxel-level FWE-corrected** ***P*** **-value within ROI** ^**b**^
	***x***	***y***	***z***		
	Carriers > non-carriers				
Right hippocampus	30	−33	−5	2.65	0.07
	Non-carriers > carriers				
Right superior/middle frontal gyrus	30	14	37	3.34	0.09

^a^MNI, Montreal Neurological Institute; ROI, Region of interest. ^b^Voxel-level family-wise error (FWE)–corrected threshold of *P* < 0.05.

### Correlation between neuropsychological performance and white matter hyperintensity volume

Performance on the TMT A was similar between carriers and non-carriers of the *APOE* ε4 allele (Table 1 and Additional file 2: Table S2) and exhibited a trend-level association with total WMH volume in the group overall (in subjects with WMH volume ≤10 cm^3^: *f*(1, 164) = 3.5; *P* = 0.064). In the subgroup of ε4 non-carriers, WMH load showed a significant negative effect on TMT A performance (*f*(1, 49) = 4.6; *P* = 0.036), contrary to its effect in ε4 carriers (*f*(1, 102) = 0.32; *P* = 0.57).

### Correlation between neuropsychological performance and gray matter volume distribution

In both *APOE* subgroups, performance on the TMT A correlated with GM volume in the parietal and temporal regions; ε4 carriers also showed effects in frontal cortex (Table 5, Figure 3C and 3D).

**Table 5:** **Correlations between gray matter volume and Trail Making Test A performance in** ***APOE*** ε**4 carriers and non-carriers**

**Location**	**MNI coordinates**			**z-value**	**Cluster-level FWE-corrected** ***P*** **-value**
	***x***	***y***	***z***		
	TMT A				
	*Carriers*				
Right inferior frontal gyrus	38	22	16	4.28	0.01
Right precuneus	14	−51	25	3.48	0.01
Right supramarginal gyrus	57	−34	33	4.47	0.001
Left supramarginal gyrus	−30	−46	37	4.37	0.01
Right superior temporal gyrus	46	−27	15	4.15	0.001
Right middle temporal gyrus	51	−61	−9	3.98	0.01
Left middle temporal gyrus	58	−19	−8	3.47	0.01
Right inferior temporal gyrus	40	−45	−27	4.54	0.001
Left inferior temporal gyrus	−34	−54	−9	4.02	0.01
Right fusiform gyrus	32	−61	−5	4.13	0.001
Left fusiform gyrus	−32	−52	−9	4.07	0.01
	*Non-carriers*				
Right precuneus	8	−48	43	4.31	0.01
Right supramarginal gyrus	39	−34	37	3.36	0.01
Left supramarginal gyrus	−44	−31	24	3.53	0.05
Right angular gyrus	54	−54	33	3.89	0.05
Left angular gyrus	−42	−42	42	3.65	0.05
Right superior parietal lobule	39	−58	54	3.42	0.05
Right inferior parietal lobule	44	−52	48	3.39	0.05
Right middle temporal gyrus	54	−55	24	3.45	0.05
Left middle temporal gyrus	−44	−45	12	3.98	0.05
	Delayed verbal recall				
	*Carriers*				
Right hippocampus	36	−30	−8	2.65	0.08^b^
Left hippocampus	−33	−34	−8	3.49	0.01^b^
	*Non-carriers*				
	–	–	–	–	–

^a^MNI, Montreal Neurological Institute. ^b^Voxel-level family-wise error (FWE)–corrected threshold *P*-values within region of interest.

Delayed verbal recall performance correlated with hippocampal volume in the group of ε4 carriers (Table 5, Figure 3E). There were no correlations between recall performance and GM volume in the group of non-carriers.

## Discussion

In the present study, we provide evidence that WM damage in patients with AD is more pronounced in non-carriers of the *APOE* ε4 allele than in carriers. Though ε4 carriers and non-carriers were well matched for disease severity, WMH volume was greater and showed a cognitive impact in the group of non-carriers. Voxel-based permutation testing confirmed greater periventricular WMH volume in non-carriers, in line with the observed difference in global lesion load and a periventricular focus of WMHs in both *APOE* subgroups. These findings are consistent with WM lesion mechanisms of structural damage and cognitive impairment in AD that complement those related to *APOE* genetic risk.

### Cognitive impact of white matter hyperintensities

In the group of *APOE* ε4 non-carriers, correlations occurred between global WMH volume and performance on the TMT, which is sensitive to deficits of attention, executive function and speed of information processing [46]. In elderly subjects, associations of impairment in these domains with WMHs have frequently been reported [21,34,47-50]. Though strategic locations for lesions linked to reduced processing speed and executive deficits have recently been identified in WM as well as in subcortical structures [34,47], these are widely distributed, indicating complex network demands [51] as well as susceptibility to small-vessel ischemic disease.

The observed contribution of WMHs to cognitive impairment in the group of ε4 non-carriers is consistent with cerebrovascular pathology frequently found in AD [52]. Whether microvascular disease is, in fact, more strongly associated with cognitive decline in non-carriers of the ε4 allele needs to be confirmed in longitudinal studies. Because of the more extensive WMH load, retrograde and downstream neuronal damage resulting from axonal injury are also likely accountable for substantial GM atrophy in this subgroup [29,53,54].

Of note, frontal atrophy was associated with impaired TMT performance only among carriers, whereas temporal and parietal GM volume effects were identified in both groups. The additional involvement of the frontal cortex in ε4 carriers may reflect a closer link between cortical AD pathology and cognitive performance in this subgroup, but it could also be related to the difference in group size.

Contrary to speed of information processing and executive function as tested by the TMT, verbal delayed recall was not associated with WMH load in either group, and it showed greater impairment in carriers of the *APOE* ε4 allele. Furthermore, delayed recall performance in the group of ε4 carriers correlated with hippocampal volume, which, in turn, exhibited a trend toward more pronounced atrophy. The detection of reduced episodic memory performance and hippocampal volume in *APOE* ε4 carriers compared with non-carriers, which corresponds to a pattern recently reported in a largely overlapping sample of patients with AD [55], confirms a phenotype previously identified in AD [43]. In contrast, non-carriers of the *APOE* ε4 allele with AD have been found to exhibit more pronounced executive dysfunction and more frontoparietal atrophy [43]. Of note, more accentuated executive deficits and frontoparietal atrophy were also recently reported in subjects with MCI before conversion to Alzheimer’s dementia [44], and 16 of these subjects overlapped with the sample of 201 patients with AD (8%) in our present study. Though more accentuated executive deficits were not apparent in the current group of non-carriers, possibly because performance on the easier version of the TMT A was evaluated with limited sensitivity to executive deficits, a trend toward reduced prefrontal GM volume occurred, in accord with a previously established structural phenotype [43,44].

The compatibility of *APOE*-dependent effects on GM volume detected in this study with previous findings in patients with AD with established CSF amyloid pathology [43] suggests that the sample of patients in our present study was representative of the AD population. In the absence of CSF or positron emission tomography (PET) data on amyloid pathology, the restriction to patients without strategic lesions or high volume of WMHs (>10 cm^3^; that is, Fazekas grade 3 (see Methods section)) helped exclude patients with vascular dementia in our sample, though it is still conceivable that some patients without AD pathology were included (see Limitations subsection below).

### Mechanisms of tissue damage

Carriers of the ε4 allele exhibited lower WMH volume than non-carriers, but they also showed a trend toward more prominent hippocampal atrophy, which is an early focus of AD pathology [56]. ApoE functions as a transport protein for lipids and contributes to the maintenance and repair of cell membranes, but the ε4 isoform increases the propensity of Aβ as well as neurofibrillary tangles to be deposited in the brain and reduces Aβ efflux [57,58]. Selective hippocampal vulnerability in AD has been related to its cellular architecture, specifically to synaptic subtype (for example, see [59,60]). Moreover, the hippocampus is part of the so-called default network and thus exhibits a high resting-state metabolism, which promotes the deposition of Aβ [61,62]. In ε4 carriers without cognitive deficits or with MCI, a compensatory increase in hippocampal neuronal activity and an abnormally high metabolism in this region have been detected, the latter of which is likely to accelerate Aβ aggregation [61,62]. Thus, the *APOE* ε4 allele may predispose individuals toward the mediotemporally focused pattern of neurodegeneration typically associated with AD [56].

Conversely, the high WMH load in non-carriers of the ε4 allele may mirror a pathogenetic mechanism necessary to develop AD in the absence of *APOE* ε4-mediated neurodegeneration. This may be a cumulative effect of atherosclerosis induced by non-specific vascular risk factors and also of AD pathology, but it is also likely a reflection of convergent processes [2-4,63-65]. Evidence has accumulated that a range of factors, such as blood pressure, lipid metabolism and insulin sensitivity, influence levels of amyloid and neurofibrillary deposition and may affect endothelial integrity [63,65-68].

Arterial hypertension, specifically increased diastolic blood pressure, was associated with WMH volume in our sample. Thus, it is conceivable that WMHs in the present study indicate synergistic adverse effects of elevated diastolic blood pressure and amyloid-mediated endothelial damage. Amyloid is known to accumulate in blood vessels, as well as in the brain parenchyma, and thus likely to confer an increased endothelial vulnerability to hypertension [7]. Conversely, amyloid deposition may compound endothelial damage induced by hypertension [66].

WMH may also, at least in part, indicate an additional factor that lowers the threshold for Alzheimer’s dementia [17]. In accordance with this notion, a recent investigation showed an association of WMHs with several vascular risk factors, such as high blood pressure, but not with CSF levels of Aβ_42_, in patients with probable AD. In contrast, WMH microbleeds visualized on T2*-weighted MRI scans were linked to arterial hypertension as well as to low levels of CSF Aβ_1–42_ and homozygosity for the *APOE* ε4 allele [63]. Moreover, WMH and amyloid positivity based on PET data have been shown to contribute independently to AD risk [17].

Interestingly, subjects without dementia who have the *APOE* ε4 allele have been shown to exhibit increased WMH volume [69,70]. In contrast, ε4 non-carriers had greater WMH accumulation in our present sample of patients with AD. A possible explanation for this apparent disparity may be that ε4 carriers are generally at increased risk of developing amyloid-induced endothelial damage, but that AD in the absence of the ε4 risk allele is, to a considerable degree, based on substantial and presumably multifactorial WM injury.

In ε4 non-carriers, a greater complexity or a different emphasis of factors may confer vulnerability to microvascular damage [71,72]. Recently, genetic variants conferring risk of WMHs in subjects without dementia, stroke or clinical cardiovascular disease have become a focus of genome-wide association studies. A meta-analysis revealed six novel single-nucleotide polymorphisms in one locus on chromosome 17q25 related to WMH burden [72]. In patients with AD, genetic variants conveying risk of WMHs may interact with AD susceptibility genes.

Lesion distribution showed periventricular foci in carriers and non-carriers of the ε4 allele, again suggesting a convergence of pathological pathways. Ischemic lesions tend to develop in periventricular watershed areas perfused by subependymal arteries with few anastomoses, as well as in subcortical regions, indicating fiber loss secondary to ischemia [7,71]. WM areas especially vulnerable to amyloid deposition appear to be in the posterior periventricular region, which is also susceptible to confluent ischemic lesions [7]. The effect seen in the corpus callosum is less compatible with ischemic damage and/or amyloid-mediated vascular injury and may in part reflect low interindividual variability and thus high statistical power compared with other locations (see the study limitations described below).

### Limitations

Because data were gathered at several sites, it cannot be excluded that differences in MRI hardware and protocols lowered the sensitivity for volume effects. To control for center effects, center affiliations were used as covariates. However, some brain areas, particularly along the midsagittal plane, may be especially sensitive to scanning parameters [73]. Voxel-based lesion symptom mapping has the general limitation that a minimum number of voxels in a specific location are required to perform robust group analyses. Individual variability in lesion location, and thus in statistical power, are likely to vary regionally [42,74]. Thus, effects in peripheral locations may have been missed because lesions showed insufficient overlap.

Furthermore, the participants of this cohort were relatively homogeneous, which may limit the generalizability of the results. Because the diagnosis of AD was based on clinical criteria in the present study, as opposed to CSF- or PET-based evidence, the restriction to patients without strategic vascular lesions or severe WMH volume was important to help exclude patients with vascular dementia. Nevertheless, it cannot be excluded that there were more subjects with vascular dementia in the group of *APOE* ε4 non-carriers than among carriers, also considering that mean diastolic blood pressure and frequency of antihypertensive medication use were slightly, though not significantly, higher among ε4 non-carriers (Table 1 and Additional file 2: Table S2). To limit the impact of vascular risk factors on the observed group difference in WMH volume, we controlled for blood pressure, diabetes and coronary heart disease, as well as antihypertensive and cholesterol-lowering treatment, and we found that the *APOE*-dependent effect on WMH volume persisted.

Recently, altered CNS insulin signaling associated with reduced cerebral insulin receptor density has emerged as a pathogenic factor in AD that may be modulated by the *APOE* genotype [75,76]. In the present study, data on CNS and peripheral insulin sensitivity were not available. Diabetes, which only affected a small number of patients, was not linked to increased WMH volume or to *APOE* genotype. Considering that peripheral insulin resistance has been reported to correlate with WMH load in subjects without diabetes [77] and that the impact of CNS insulin resistance on WM integrity is not known, meaningful associations between insulin resistance, WM damage and *APOE* genotype may have remained undetected here and should be addressed in future investigations.

## Conclusions

Our finding of an *APOE*-dependent effect on WMH load suggests a more prevalent and functionally relevant contribution of WMHs to cognitive impairment in AD among *APOE* ε4 non-carriers. Thus, an increased prevalence of WMHs may reflect a complementary structural pathway of progression to dementia. The observed effects of *APOE* risk allele as well as hypertension on WMH volume emphasize the importance of attending to microvascular pathology in AD, which so far has frequently been an exclusion criterion in AD studies. This may also help to refocus clinical efforts on cerebrovascular damage in AD. To further elucidate the role of WMHs in AD, future studies will need to include CSF or PET markers of AD pathology, peripheral and CNS measures of insulin sensitivity, and additional genetic risk variants. Because antihypertensive drugs may have differential effects on the incidence and progression of AD via their impact on the metabolism of Aβ in the brain [78,79], type of antihypertensive medication needs to be considered in future studies. Longitudinal investigations are necessary to indicate the dynamics of WM damage in AD. Ultimately, a more profound understanding of heterogeneous disease mechanisms in AD may facilitate more targeted therapeutic approaches.

## Additional files

Additional file 1: Table S1. Characteristics of patients excluded because of WMH volume >10 cm^3^ (n = 18). Additional file 2: Table S2. Characteristics of patients including subjects with severe WMH volume (n = 201). Additional file 3: Table S3. Effects on total WMH volume (analysis of variance model with *APOE* ε4 status (ε4 non-carrier versus carrier) as factor of interest) including subjects with WMH volume >10 cm^3^. Additional file 4: Table S4. Effects on total WMH volume (analysis of variance model with dose of *APOE* ε4 alleles as factor of interest).

## Abbreviations

Aβ: Amyloid-β
AD: Alzheimer’s disease
ANOVA: Analysis of variance
ApoE/*APOE*: Apolipoprotein E
CDR: Clinical Dementia Rating
CDR SOB: Clinical Dementia Rating Sum of Boxes
CNS: Central nervous system
CSF: Cerebrospinal fluid
FLAIR: Fluid-attenuated inversion recovery
FEW: Family-wise error
GM: Gray matter
IRB: Institutional review board
LST: Lesion Segmentation Toolbox
MCI: Mild cognitive impairment
MMSE: Mini Mental State Examination
MNI: Montreal Neurological Institute
MRI: Magnetic resonance imaging
NINCDS-ADRDA: National Institute of Neurological and Communicative Disorders and Stroke–Alzheimer’s Disease and Related Disorders Association
NINDS-AIREN: National Institute of Neurological Disorders and Stroke–Association Internationale pour la Recherche et l’Enseignement en Neurosciences
NPI: Neuropsychiatric Inventory
PET: Positron emission tomography
ROI: Region of interest
SD: Standard deviation
SE: Standard error
TE: Echo time
TR: Repetition time
TMT: Trail Making Test
VBM: Voxel-based morphometry
WM: White matter
WMH: White matter hyperintensities
