# Supplementary material for: Apolipoprotein E-dependent load of white matter hyperintensities in Alzheimer’s disease: a voxel-based lesion mapping study
Source: Alzheimers Res Ther. 2015 May 15;7(1):27. doi: 10.1186/s13195-015-0111-8 (PMC4432954; doi:10.1186/s13195-015-0111-8)
Supplement: Additional file 1: Table S1. — Characteristics of patients excluded because of WMH volume >10 cm3 (n = 18). [file 13195_2015_111_MOESM1_ESM.doc]

Additional file 1 Table S1: Characteristics of patients excluded because of WMH volume >10 cm^3^ (n=18)

|  | APOE ε-4 carriers  (n=8) | APOE ε-4 non-carriers  (n=10) |
| --- | --- | --- |
|  | Mean + SD or ratio | Mean + SD or ratio |
| Age (years) | 75.3 + 5.2 | 81.4 + 4.7 |
| Age of onset (years) | 72.4 + 7.0 | 78.0 + 2.8 |
| Duration of disease (months) | 31.7 + 25.6 | 20.0 + 17.0 |
| Gender (men/women) | 2/6 (1 : 3) | 5/5 (1 : 1) |
| Education (yrs.) | 8.5 + 0.9 | 9.9 + 2.0 |
| Total lesion volume (mm^3^) | 14688 + 4155 | 17960 + 8867 |
| Systolic blood pressure (mm Hg) | 153.3 + 31.9 1 | 150.6 + 21.5 2 |
| Diastolic blood pressure (mm Hg) | 86.5 + 6.3 1 | 79.9 + 7.4 2 |
| Antihypertensive medication (yes/no) | 4/4 | 5/5 |
| Coronary heart disease (yes/no) | 2/6 | 2/7^†^ |
| Diabetes (yes/no) | 3/5 | 2/8 |
| Hypercholesterolemia (yes/no) | 3/5 | 2/6^‡^ |
| BMI | 21.4 + 3.1 | 26.4 + 3.3^†^ |
| CDR SOB | 4.6 + 0.7 | 4.7 + 2.0 |
| MMSE (score) | 22.9 + 2.0 | 23.5 + 3.2 |
| Delayed verbal recall (score)^*^ | 1.5 + 1.1 | 1.6 + 2.0 |
| Verbal learning | 11.5 + 5.1 | 12.4 + 4.4 |
| Trail Making Test A (s) | 124.0 + 74.4 | 97.3 + 58.8 |
| Constructive Praxia | 7.9 + 2.4 | 9.0 + 1.2 |
| Boston Naming Test | 11.9 + 1.8 | 12.3 + 1.7 |

Available data in: ^†^n=9, ^‡^n=8
