# Supplementary material for: Apolipoprotein E-dependent load of white matter hyperintensities in Alzheimer’s disease: a voxel-based lesion mapping study
Source: Alzheimers Res Ther. 2015 May 15;7(1):27. doi: 10.1186/s13195-015-0111-8 (PMC4432954; doi:10.1186/s13195-015-0111-8)
Supplement: Additional file 2: Table S2. — Characteristics of patients including subjects with severe WMH volume (n = 201). [file 13195_2015_111_MOESM2_ESM.doc]

Additional file 2: Table S2: Characteristics of patients including subjects with severe WMH volume (n=201)

|  | APOE ε4 carriers  (n=128) | APOE ε4 non-carriers  (n=73) | Group comparison |
| --- | --- | --- | --- |
|  | Mean + SD or ratio | Mean + SD or ratio | p |
| Age (years) | 70.7 + 6.5 | 71.8 + 9.0 | 0.32 |
| Age of onset (years) | 68.0 + 7.2 | 68.2 + 12.0 | 0.34 |
| Duration of disease (months) | 31.3 + 24.6 | 29.1 + 24.6 | 0.55 |
| Gender (men/women) | 59/69 (1 : 1.2 ) | 33/40 (1 : 1.2 ) | 0.90 |
| Education (yrs.) | 9.0 + 1.8 | 9.5 + 2.1 | 0.12 |
| Systolic blood pressure (mm Hg) | 139.9 + 17.7^‡^ | 142.3 + 17.6^§^ | 0.45 |
| Systolic blood pressure > 140 mm Hg (yes/no) | 60/43 (1.4 : 1) | 38/25 (1.5 : 1) | 0.89 |
| Diastolic blood pressure (mm Hg) | 81.8 + 8.9^‡^ | 83.3 + 7.9^§^ | 0.28 |
| Diastolic blood pressure > 90 mm Hg (yes/no) | 34/69 (1 : 2) | 21/42 (1 : 2) | 0.96 |
| Antihypertensive medication (yes/no) | 35/91 (1 : 2.6)^¶^ | 25/44 (1 : 1.8)^#^ | 0.29 |
| Coronary heart disease (yes/no) | 9/117 (1 : 13)^§^ | 7/63 (1 : 9)** | 0.85 |
| Diabetes (yes/no) | 14/102 (1 : 7.3)^¶^ | 5/58 (1 : 11.6)^¶^ | 0.45 |
| Hypercholesterolemia (yes/no) | 16/107 (1: 6.7)^††^ | 9/53 (1 : 5.9)^‡‡^ | 0.77 |
| BMI | 24.4 + 3.9^‡^ | 24.5 + 4.7^§^ | 0.45 |
| CDR SOB | 4.3 + 1.3 | 4.3 + 1.6 | 0.82 |
| MMSE (score) | 23.7 + 3.1^¶¶^ | 24.4 + 2.9 72^††^ | 0.64 |
| Delayed verbal recall (score)^*^ | 2.0 + 2.0^¶¶^ | 2.7 + 2.2 | *0.03* |
| Verbal learning | 12.1 + 4.2^¶¶^ | 12.2 + 4.4 | 0.90 |
| Trail Making Test A (s) | 98.4 + 58.3^##^ | 96.3 + 52.8 | 0.57 |
| Constructive Praxia | 9.0 + 2.3^¶¶^ | 9.0 + 1.7 | 0.75 |
| Boston Naming Test | 12.7 + 2.1^¶¶^ | 12.7 + 2.3 | 0.88 |

P-Values are based on t-tests.

Available data in: ^‡^n=103, ^§^n=63, ^¶^n=126, ^#^n=69, **n=70, ^††^n=123, ^‡‡^n=62, ^§§^n=99, ^¶¶^n=127, ^##^n=124
