# Supplementary material for: Apolipoprotein E-dependent load of white matter hyperintensities in Alzheimer’s disease: a voxel-based lesion mapping study
Source: Alzheimers Res Ther. 2015 May 15;7(1):27. doi: 10.1186/s13195-015-0111-8 (PMC4432954; doi:10.1186/s13195-015-0111-8)
Supplement: Additional file 3: Table S3. — Effects on total WMH volume (analysis of variance model with APOE ε4 status (ε4 non-carrier versus carrier) as factor of interest) including subjects with WMH volume >10 cm3. [file 13195_2015_111_MOESM3_ESM.doc]

| Additional file 3: Table S3: Effects on total WMH volume (analysis of variance model with APOE ε4 status [ε4 non-carrier vs. carrier] as factor of interest) including subjects with WMH volume >10 cm^3^ | | | | |
| --- | --- | --- | --- | --- |
|  | Model 1 (n = 201) | | Model 2 (n = 138) | |
|  | F[1,186] | P | F[1,115] | p |
| **APOE ε4 carrier status** | **9.3** | **0.01** | 6.9 | 0.01 |
| Age | 38.045 | 0.0001 | 22.5 | 0.0001 |
| Sex | 0.008 | 0.93 | 0.47 | 0.50 |
| Education | 0.01 | 0.98 | 0.11 | 0.74 |
| Disease status | 0.953 | 0.33 | 0.58 | 0.45 |
| Total intracranial volume | 0.206 | 0.65 | 1.3 | 0.25 |
| Duration of disease |  |  | 1.1 | 0.29 |
| MMSE score |  |  | 0.39 | 0.53 |
| Systolic blood pressure |  |  | 1.19 | 0.28 |
| **Diastolic blood pressure** |  |  | 0.14 | 0.71 |
| Antihypertensive medication |  |  | 0.08 | 0.79 |
| Coronary heart disease |  |  | 0.13 | 0.72 |
| Cholesterol medication |  |  | 0.08 | 0.77 |
| Diabetes |  |  | 0.24 | 0.62 |
| Results for site covariates are not reported | | |  |  |
