# Supplementary material for: Apolipoprotein E-dependent load of white matter hyperintensities in Alzheimer’s disease: a voxel-based lesion mapping study
Source: Alzheimers Res Ther. 2015 May 15;7(1):27. doi: 10.1186/s13195-015-0111-8 (PMC4432954; doi:10.1186/s13195-015-0111-8)
Supplement: Additional file 4: Table S4. — Effects on total WMH volume (analysis of variance model with dose of APOE ε4 alleles as factor of interest). [file 13195_2015_111_MOESM4_ESM.doc]

| Additional file 4: Table S4: Effects on total WMH volume (analysis of variance model with dose of APOE ε4 alleles as factor of interest) | | | | |
| --- | --- | --- | --- | --- |
|  | Model 1 (n = 183) | | Model 2 (n = 129) | |
|  | F[1,169] | P | F[1,107] | p |
| **APOE ε4 allele dose** | **4.7** | **0.01** | **4.7** | **0.01** |
| Age | 39.9 | 0.01 | 42.6 | 0.01 |
| Sex | 1.4 | 0.25 | 2.3 | 0.13 |
| Education | 0.46 | 0.50 | 0.01 | 0.91 |
| Disease status | 1.37 | 0.24 | 1.7 | 0.20 |
| Total intracranial volume | 4.5 | 0.04 | 8.8 | 0.004 |
| Duration of disease |  |  | 0.09 | 0.77 |
| MMSE score |  |  | 0.01 | 0.93 |
| Systolic blood pressure |  |  | 0.2 | 0.68 |
| **Diastolic blood pressure** |  |  | **10.4** | **0.002** |
| Antihypertensive medication |  |  | 0.4 | 0.55 |
| Coronary heart disease |  |  | 0.002 | 0.97 |
| Cholesterol medication |  |  | 0.4 | 0.55 |
| Diabetes medication |  |  | 0.02 | 0.89 |
| Results for site covariates are not reported | | |  |  |
